# Macrophage‐derived MMP‐9 enhances the progression of atherosclerotic lesions and vascular calcification in transgenic rabbits

**DOI:** 10.1111/jcmm.15087

**Published:** 2020-03-03

**Authors:** Yajie Chen, Ahmed Bilal Waqar, Kazutoshi Nishijima, Bo Ning, Shuji Kitajima, Fumikazu Matsuhisa, Lu Chen, Enqi Liu, Tomonari Koike, Ying Yu, Jifeng Zhang, Yuqing Eugene Chen, Huijun Sun, Jingyan Liang, Jianglin Fan

**Affiliations:** ^1^ Department of Molecular Pathology Faculty of Medicine Graduate School of Medical Sciences University of Yamanashi Yamanashi Japan; ^2^ Bioscience Education‐Research Support Center Akita University Akita Japan; ^3^ School of Biotechnology and Health Sciences Wuyi University Jiangmen China; ^4^ Analytical Research Center for Experimental Sciences Saga University Saga Japan; ^5^ Research Institute of Atherosclerotic Disease and Laboratory Animal Center Xi'an Jiaotong University School of Medicine Xi'an China; ^6^ Center for Advanced Models for Translational Sciences and Therapeutics University of Michigan Medical Center Ann Arbor MI USA; ^7^ Department of Pharmacology Dalian Medical University Dalian China; ^8^ Research Center for Vascular Biology School of Medicine Yangzhou University Yangzhou China

**Keywords:** atherosclerosis, calcification, hypercholesterolaemia, macrophage, MMP‐9, transgenic rabbits

## Abstract

Matrix metalloproteinase‐9 (MMP‐9), or gelatinase B, has been hypothesized to be involved in the progression of atherosclerosis. In the arterial wall, accumulated macrophages secrete considerable amounts of MMP‐9 but its pathophysiological functions in atherosclerosis have not been fully elucidated. To examine the hypothesis that macrophage‐derived MMP‐9 may affect atherosclerosis, we created MMP‐9 transgenic (Tg) rabbits to overexpress the rabbit MMP‐9 gene under the control of the scavenger receptor A enhancer/promoter and examined their susceptibility to cholesterol diet‐induced atherosclerosis. Tg rabbits along with non‐Tg rabbits were fed a cholesterol diet for 16 and 28 weeks, and their aortic and coronary atherosclerosis was compared. Gross aortic lesion areas were significantly increased in female Tg rabbits at 28 weeks; however, pathological examination revealed that all the lesions of Tg rabbits fed a cholesterol diet for either 16 or 28 weeks were characterized by increased monocyte/macrophage accumulation and prominent lipid core formation compared with those of non‐Tg rabbits. Macrophages isolated from Tg rabbits exhibited higher infiltrative activity towards a chemoattractant, MCP‐1 in vitro and augmented capability of hydrolysing extracellular matrix in granulomatous tissue. Surprisingly, the lesions of Tg rabbits showed more advanced lesions with remarkable calcification in both aortas and coronary arteries. In conclusion, macrophage‐derived MMP‐9 facilitates the infiltration of monocyte/macrophages into the lesions thereby enhancing the progression of atherosclerosis. Increased accumulation of lesional macrophages may promote vascular calcification.

## 1 INTRODUCTION

Matrix metalloproteinases (MMPs), first described in 1962,^1^ are a family of zinc‐dependent endopeptidases with more than 23 kinds reported in humans.^2^ MMPs play crucial roles in several physiological processes, including tissue remodelling, immune functions, reproduction and development, but are also involved in many pathological processes, such as tumour invasion, autoimmune diseases, neurodegenerative disorders and cardiovascular diseases.^3^, ^4^, ^5^ MMPs are roughly divided into two major types according to their molecular properties: membrane‐anchored and secreted. Furthermore, based on the hydrolysis substrates, secreted‐type MMPs can be further classified into collagenases, gelatinases, stromelysins and matrilysins.^5^, ^6^, ^7^ In addition to their enzymatic activities, MMPs also exhibit a number of other pathophysiological functions independent upon their ECM hydrolysis.^8^, ^9^, ^10^

The gelatinases, including MMP‐2 (72‐kD) and MMP‐9 (92‐kD), are different from other MMPs due to a collagen‐binding domain within the catalytic domain that is involved in the binding of collagenous substrates, elastin and thrombospondins.^11^ MMP‐9, also called gelatinase B, was first discovered as an enzyme involved in extracellular matrix (ECM) remodelling by degradation of denatured collagens (gelatins).^12^ In the arterial walls, MMP‐9 is synthesized and secreted by endothelial cells, smooth muscle cells as well as macrophages and is involved in the regulation of cell survival, migration, inflammation and angiogenesis.^2^, ^13^ Increased MMP‐9 activity is associated with several diseases such as asthma,^14^ systemic lupus erythematosus,^15^ abdominal aortic aneurysms,^16^ plaque rupture,^17^ left ventricular hypertrophy^18^ and stroke.^19^

Accumulation of monocytes and macrophage‐derived foam cells in the intima of large arteries is a hallmark of human and experimental animal atherosclerosis.^20^ Macrophages and macrophage‐derived foam cells in the arterial wall secrete substantial amounts of MMP‐9, in concert with other MMPs, which have been considered to participate in the pathogenesis of atherosclerosis and plaque rupture.^17^, ^21^, ^22^ However, it is not clear whether macrophage‐derived MMP‐9 is directly involved in the initiation or progression or both of atherosclerosis although MMP‐9 was detected in the lesions of human atherosclerosis.^23^, ^24^ Functional polymorphism in the regulatory region of MMP‐9 has been found to be associated with the severity of coronary atherosclerosis,^25^ whereas high levels of plasma MMP‐9 protein concentrations are closely correlated with acute coronary syndrome.^26^, ^27^ However, conflicting results have been reported in terms of MMP‐9 function in the pathogenesis of atherosclerosis using apoE knockout (KO) mice. In one report, MMP‐9 deficiency protected against cholesterol diet‐induced atherosclerosis,^28^ but in another, MMP‐9 inactivation increased the atherosclerotic plaque growth and progression.^29^ In addition, a report indicated that overexpressing human MMP‐9 in macrophages increased collagen content in lesions but showed no effects on atherosclerotic lesions in apoE KO mice.^30^ Recently, we performed an RNAseq analysis of the aortic lesions of both cholesterol‐fed and WHHL rabbits and showed that MMP‐9 along with MMP‐1 and MMP‐12 was predominately up‐regulated compared with aortas of normal wild‐type rabbits.^31^ As MMP‐9 is able to degrade several other ECMs in the arterial wall, we hypothesized that elevation of MMP‐9 expression may participate in or mediate atherosclerotic lesion formation. To test this hypothesis, we generated transgenic (Tg) rabbits overexpressing rabbit MMP‐9 gene specifically in the macrophage lineage and foam cells of atherosclerotic lesions. The rationale of using rabbits for this undertaking is twofold. First, compared with wild‐type murine models, rabbits are more sensitive to a cholesterol diet and develop atherosclerosis rapidly. Therefore, it is possible to generate different types of atherosclerotic lesions in cholesterol‐fed rabbits. Second, atherosclerotic lesions of cholesterol‐fed rabbits are rich in macrophage‐derived foam cells, which facilitates the analysis of macrophage functions in the arterial wall.^32^ To the best of our knowledge, this is the first report to demonstrate that overexpression of MMP‐9 in macrophages is not only involved in the progression of atherosclerosis but also increases vascular calcification.

## 2 MATERIALS AND METHODS

### 2.1 Generation and characterization of Tg rabbits

**Figure 1:**
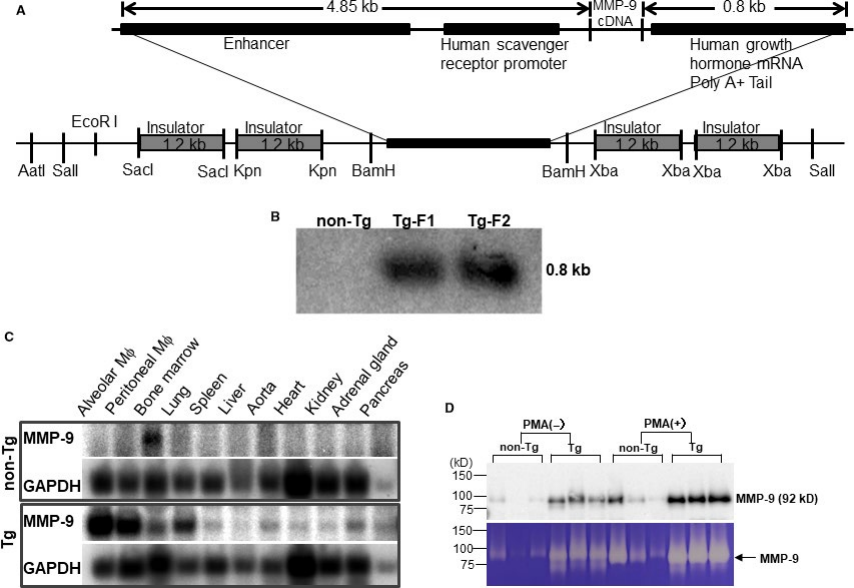
Transgenic construct for generation of transgenic rabbits. The DNA construct used for microinjection was composed of rabbit MMP‐9 cDNA under the control of a human scavenger receptor A enhancer/promoter region (5 kb) along with four copies of the chicken β globin insulator (A). Two Tg founders (designated as F1 and F2) were identified by Southern blot analysis using a rabbit MMP‐9 cDNA probe (B). Northern blotting analysis revealed that endogenous MMP‐9 expression was mainly in the bone marrow of non‐Tg rabbits, whereas Tg MMP‐9 was expressed in isolated macrophages, bone marrow and lungs. GAPDH was used as an internal control (C). Peritoneal macrophages were isolated from both Tg and non‐Tg rabbits (3‐mon), and incubated in serum‐free medium for 48 h with or without PMA. Conditioned media were collected and analysed for MMP‐9 protein and enzymatic activity by Western blotting and zymographic analysis. Each lane represents a sample from an individual animal and n = 3 for each group (D)

Tg rabbits were generated by the methods established in our laboratory as reported previously.^33^, ^34^ The DNA construct used for microinjection was composed of rabbit MMP‐9 cDNA under the control of the human scavenger receptor enhancer/promoter along with four copies of the chicken β globin insulator (Figure 1A), which prevents the position effect of transgene insulators.^35^, ^36^ In total, 1032 embryos were injected, and 879 embryos were implanted into 40 recipient female rabbits. Ten recipients gave birth to 26 pups, and among them, 2 pups were found to carry the transgenes by Southern blotting (Figure 1B). Because mRNA expression levels of MMP‐9 analysed by Northern blots were similar in both founders, Tg founder F1 rabbit was mated with wild‐type rabbits to produce following progeny. In this study, rabbits at the age of 4 months were used unless otherwise specified. All rabbits were fed either with a chow diet or cholesterol‐rich diet containing 0.5% cholesterol and 3% soybean oil (see below).^37^ The rabbits were allowed access to the diet and water ad libitum. Plasma levels of total cholesterol (TC), triglycerides (TG) and HDL‐cholesterol (HDL‐C) were analysed weekly using the methods described previously.^38^ All animal experiments were performed with the approval of the Animal Care Committee of the Universities of Yamanashi and Saga and conformed to the Guide for the Care and Use of Laboratory Animals published by the US National Institutes of Health.

### 2.2 Analysis of MMP‐9 expression

To examine the tissue expression of MMP‐9 in Tg and non‐Tg rabbits, Northern blotting was performed as described previously.^39^ In brief, total RNA was isolated from bone marrow, lung, spleen, liver, aorta, heart, kidney, adrenal, pancreas, and both alveolar and peritoneal macrophages (see below) using TRIzol reagent (Life Technologies, Inc) and 10 μg of RNA was denatured in the presence of dimethyl sulfoxide and glyoxal was subjected to electrophoresis in a 1.2% formaldehyde agarose gel, and then transferred to a Nitran nylon membrane and then treated with UV light in the UV crosslinker. The membrane was hybridized with the ^32^P‐labelled a rabbit MMP‐9 cDNA probe synthesized with a Prime‐It II random primer labelling kit (Stratagene). The blot was rehybridized with a ^32^P‐labelled human β‐actin probe (Clontech Laboratories) to confirm that equal amounts of RNA were loaded in each lane. To evaluate MMP‐9 protein expression and enzymatic activity, we collected elicited peritoneal macrophages from the peritoneal cavity 4 days after injection of 4% Brewer's thioglycollate broth, as described previously.^40^ In brief, rabbits were anaesthetized by intramuscular injection of ketamine (25 mg/kg BW) + medetomidine hydrochloride (0.5 mg/kg BW) and restrained with ventral side up. Thioglycollate broth loaded in 50‐mL syringes was injected into the peritoneal cavity. 4 days later, rabbits were euthanized by injection of sodium pentobarbital solution (100 mg/kg BW) through an ear vein. Abdominal cavity was cut open along the middle line and washed three times using 100 mL of phosphate‐buffered saline (pH 7.4) with heparin (10 U/L). After centrifugation, peritoneal macrophages (10 × 10^6^) from either Tg or non‐Tg rabbits (n = 5 for each group) were incubated in serum‐free 1640 medium with or without phorbol 12‐myristate 13‐acetate (Sigma‐Aldrich Com) (50 ng/mL) for 48 hours, and the conditioned media were then collected for Western blotting and gelatin zymographic analysis.^32^ The same aliquots of the conditioned media from each group were fractionated by electrophoresis on 10% SDS‐polyacrylamide gels, transferred onto a nitrocellulose membrane, then incubated with monoclonal antibody (mAb) (Table S1) against human MMP‐9, which cross‐reacted with rabbit MMP‐9. To evaluate gelatinase activity, we first performed gelatin gel zymographic analysis using the method reported previously.^32^ Briefly, a piece of fresh aortic arch was homogenized in ice‐cold RIPA. 20 μg proteins were separated by electrophoresis through 8% SDS‐polyacrylamide gels containing gelatin (1 mg/mL) (Wako Pure Chemical Industries Ltd., Osaka, Japan) under non‐denaturing and non‐reducing conditions. After washing in 2.5% Triton X‐100 (Sigma‐Aldrich) to remove SDS, the gels were incubated in buffer solution (50 mmol/L Tris‐HCl, pH = 7.5, 10 mmol/L CaCl_2_, 1 μmol/L ZnCl_2_, 0.2 mmol/L NaN_3_ and 0.05% BRIJ‐35) at 37°C for 36 hours. After that, the gels were stained by Coomassie Brilliant Blue (Wako) till the bands were visualized. Areas of enzymatic activity showed as clear bands over the mazarine background. Gels were scanned, and the density of digested bands was quantified with ImageJ software (National Institutes of Health). To measure the enzymatic activity of gelatinases in the aortic tissue directly, we examined gelatin activity of aortic arch homogenate (n = 4 for each group) with an EnzChek Gelatinase kit (Molecular Probes, Eugene, Oregon). 100 μg of crude protein of each sample was incubated with quenched fluorescein‐conjugated gelatin at 37°C in darkness. Active enzymes cleave the fluorescent‐labelled gelatin and the rate of proteolysis was expressed by fluorescence intensity. To verify the gelatinase activity specificity, aortic arch homogenate was incubated with MMP‐2/9 inhibitor (10 μmol/L) (SB‐3CT, Santa Cruz Biotechnology, Inc) for 1 hour at 37°C before mixed with quenched fluorescein‐conjugated gelatin. Fluorescence was measured at multiple time‐points during reaction using a SpectraMax Microplate Reader (Molecular Devices) (absorption 490 nm/emission 515 nm). We also performed in situ gelatinolytic activity using frozen sections of aortic arch as previously reported. 8‐µm‐thick cryosections were air‐dried for 10 minutes. After that, sections were washed with PBS to remove traces of OCT. Quenched fluorescein‐conjugated gelatin (Molecular Probes) was used as the substrate. The gelatin (20 µg/mL), which was dissolved in reaction buffer with 10% low gelling agarose (Sigma‐Aldrich), was dropped on the top of sections and incubated with reaction buffer in a dark humidity chamber at 37°C for 6 hours. 5 mmol/L EDTA in reaction buffer was used to block gelatinase activity. The sections were then rinsed with PBS and stained with DAPI for staining nuclei. Slides were then mounted with a cover glass using an aqueous agent.

### 2.3 Experimental design

Tg rabbits along with sex‐ and age‐matched non‐Tg littermates were used for the following experiment. In the first experiment, rabbits (male group: n = 6 for non‐Tg and n = 7 for Tg, female group: n = 3 for non‐Tg and n = 7 for Tg) were fed a diet containing 0.5% cholesterol and 3% soybean oil for 16 weeks. The lesions of atherosclerosis formed in these rabbits were mainly those of so‐called early‐stage lesions (fatty streaks), and thus, it was amenable to evaluate the effects of increased MMP‐9 expression on the initiation of atherosclerosis. In the second experiment, rabbits (male group: n = 10 for non‐Tg and n = 12 for Tg, female group: n = 10 for non‐Tg and n = 16 for Tg) were fed the same cholesterol‐rich diet for 28 weeks. Long‐duration hypercholesterolaemia in the second experiment in comparison with the first experiment was expected to induce more advanced atherosclerotic lesions, such as fibrous plaques and complicated plaques, in these rabbits.^41^, ^42^ Therefore, we were able to address the influence of MMP‐9 expression on the progression of atherosclerosis.

### 2.4 Analysis of aortic atherosclerosis

At the end of cholesterol diet feeding, all rabbits were euthanized by injection of sodium pentobarbital solution (100 mg/kg BW) through an ear vein. The aortas were *en face* stained by Sudan IV solution for quantitative analysis of the gross atherosclerotic lesion area as described previously.^43^ For microscopic quantification of the lesion area, each segment of the aorta arch from all rabbits was cut into cross sections as reported previously.^37^, ^44^ Then, all specimens were embedded in paraffin and sections (3 μm) were stained with haematoxylin and eosin (HE) and elastica van Gieson (EVG). For further microscopic evaluation of cellular components and MMP‐9 expression in the lesions, serial paraffin sections of the aorta arch were immunohistochemically stained with mAbs against rabbit macrophages (RAM11), α‐smooth muscle actin (HHF35), MMP‐9 and caspase‐3 (Table S1). The following antigen retrieval method was used. Citrate buffer was prepared by mixing 0.1 mol/L citric acid with 0.1 mol/L sodium citrate hydrate solution as 1:4. Then, paraffin‐embedded section slides were immersed in the citrate buffer and autoclaved at 120°C for 10 minutes. After that, slides were washed with PBS once and blocked with 10% goat serum at room temperature for 30 minutes. Abs were diluted in 10% goat serum, and slides were incubated with each first Ab at 4°C for overnight and followed by peroxidase‐conjugated goat‐antimouse IgG (Histofine Sab‐Po(M), Nichirei Bioscience, Inc) for 1 hour at room temperature. Amino‐9‐ethylcarbazole (AEC) (Nichirei Bioscience) was used as a substrate for visualizing the antigen signals and nuclei were stained with haematoxylin. To evaluate Ab specificity, the slides were incubated with mouse non‐specific IgG or PBS to replace the first Ab (Figures S11 and S12). Aortic lesions were histologically classified into early‐stage lesions (type II lesions: either fatty streaks with foam cells > 60% of the lesions or fibrotic lesions mainly composed by SMCs and ECM with foam cells < 50%) or advanced lesions (type IV atheroma or V fibroatheroma containing typical lipid or necrotic cores with calcification) according to the AHA classification.^45^ The lengths of each lesion on each section were measured and quantified as reported previously.^46^ In addition, the severity of the aortic calcification was evaluated by measuring the calcification area on each section of the aortic arch based on von Kossa staining. All section images for microscopic quantification were taken with an Olympus BX51 light microscope equipped with a DP70 digital camera (Olympus) and quantified with Lumina Vision V2.04 image analysis software (Mitani Co.). For this undertaking, we defined a colour pixel threshold of immunostaining intensity to detect the AEC‐stained red colour by selecting areas first, and then, we used the same threshold to measure colour intensity in each specimen. For analysis of Mϕ, SMC and calcification in cellular distribution, we measured and showed the real positive area. Atherosclerotic quantification was performed by two independent observers blindly. Aortic lesions were also collected and homogenized for gelatinase activity and Western blotting analysis using mAbs against MMPs and tissue inhibitors of matrix metalloproteinase (TIMPs) shown inTable S1.^32^ In brief, aortic arch was homogenized in ice‐cold RIPA buffer (Thermo Fisher Scientific) supplemented with a proteinase inhibitor cocktail (1% v/v) (Sigma‐Aldrich). After centrifugation, the supernatant was collected for measuring protein content by a Bio‐Rad protein assay kit. The supernatant was aliquoted for performing Western blotting, zymography and enzymatic assay. For Western blotting analysis, equal amounts of crude protein (30 μg) were fractionated by electrophoresis on 10% SDS‐ployacrylamide gels under reducing condition. After that, the proteins were transferred to Bio‐Rad's 0.2 μm pore‐size nitrocellulose membranes and these membranes were incubated with each Ab (Table S1) at 4°C overnight, and then washed three times with PBST (0.1% Tween 20 in 1X PBS) and reacted with horseradish peroxidase‐conjugated secondary Abs, followed by enhanced chemiluminescence detection.

### 2.5 Analysis of coronary lesions

To assess coronary atherosclerosis, all hearts of both male and female rabbits of the second experiment were cut into five blocks and the block‐I, which contains the main trunk of the left coronary artery, was used for coronary lesion analysis. The whole protocol for heart sections was described in details elsewhere^42^ and also can be obtained from the Appendix A. Supplementary data published through the following website: http://www.sciencedirect.com/science/article/pii/S0163725814001855). The blocks were cut into 4 serial sections (3 μm thick) at 50 μm intervals, and in total, 4 cuts were conducted. For 4 sections collected from each cut were stained for HE, EVG, and von Kossa or immunohistochemically stained with mAb (RAM11) against macrophages. The lesion size was quantified by EVG‐stained specimens and expressed as stenosis % (lesion area/ coronary lumen area). Calcification and Mϕ areas were quantified as aortic lesion analysis described above and expressed as mm^2^.

### 2.6 TUNEL staining

To evaluate the presence of cellular apoptosis in the lesions, serial sections of paraffin‐embedded aortas were either immunohistochemically stained by Abs against macrophage, caspase‐3 or by TUNEL (TdT‐mediated dUTP nick end labelling) method using ApopTag peroxidase in situ apoptosis detection kits (S7100, Millipore). For TUNEL staining, all the procedures followed the manufacturer's instruction. Briefly, sections of the aortic arch were deparaffinized and rehydrated through xylene and graded ethanol to distilled water. Then, specimens were treated in 3.0% hydrogen peroxide in PBS solution for 5 minutes at room temperature for quenching endogenous peroxidase. Terminal deoxynucleotidyl transferase (TdT) with digoxigenin labelled deoxyuridine triphosphate was added and incubated in a humidified chamber at 37°C for 30 minutes and then washed with PBS buffer. After that, specimens were incubated with peroxidase‐conjugated anti‐digoxigenin Ab in a humidified chamber for 30 minutes at room temperature and then washed 4 times in PBS. After that, AEC (Nichirei Bioscience) was added for visualizing the nucleotides, and nuclei were stained with haematoxylin. The negative control was conducted by using PBS to replace TdT (Figure S13).

### 2.7 Tartrate‐resistant acid phosphatase (TRAP) staining

Sections of aorta arch were deparaffinized and rehydrated through graded ethanol to distilled water. The slides were then incubated with TRAP staining solution which contained 0.1 mg/mL naphthol AS‐MX (Sigma‐Aldrich) and 0.08 mg/mL fast red violet LB salt (Sigma‐Aldrich) in the presence of 8 mmol/L sodium tartrate and 30 mmol/L sodium acetate (pH 5.0) at 37°C for 2 hours. After rinsing in distilled water, the slides were mounted with aqueous mounting medium.

### 2.8 Chemotaxis analysis and carrageenan‐induced granuloma

To examine the effects of MMP‐9 enzymatic activity on macrophage migration and matrix digestion, we performed chemotaxis analysis and granuloma assays. Chemotaxis assays for alveolar macrophages isolated from Tg and non‐Tg rabbits (n = 4 for each group) were performed using Biocoat cell culture inserts coated with laminin (Becton Dickinson Labware, Bedford, MA). The lower compartments were loaded with the same medium containing human recombinant monocyte chemoattractant protein‐1 (MCP‐1) (Pepro Tech EC) at 10 ng/mL. After 48 hours of incubation (37°C, 5% CO_2_), the number of macrophages that penetrated the membranes was counted in 10 high‐power fields randomly from each well.

We generated a carrageenan‐induced granuloma model that mimics the condition of macrophages and foam cells accumulated in atherosclerosis lesions as previously reported,^40^ to compare the infiltrative and degradative functions of macrophages of Tg rabbits with those of non‐Tg rabbits. To investigate the histological features of the granuloma, the granulomatous tissues after subcutaneous injection of carrageenan solution (Sigma‐Aldrich) for 14 days were isolated and fixed in 10% buffered formalin, embedded in paraffin, and then cut into sections (3 μm). To analyse the collagen fibre content in the granuloma, the sections were stained with Masson trichrome staining. The total area of collagen staining was calculated using the image analysis system as above described.

### 2.9 Statistical analysis

Statistical analysis was performed using SPSS 16.0 software. All data are expressed as mean ± SEM. Statistical analysis was performed for parametric data by Student's *t* test and for non‐parametric data (calcification study) by the Mann‐Whitney *U* test. In all cases, significance was set as a *P*‐value <.05.

## 3 RESULTS

### 3.1 Generation of Tg rabbits

We generated 2 Tg founder rabbits as shown by Southern blotting analysis (Figure 1B). One founder rabbit was bred to provide F1 progeny for the current study. Tg rabbits did not exhibit any visible abnormalities in the whole‐body appearance and histological examinations did not reveal any pathological changes in heart, lung, spleen, adrenal and liver. Peripheral blood white cells in Tg rabbits were not different from those of non‐Tg rabbits (data not shown). Northern blotting analysis revealed that Tg rabbits expressed prominent MMP‐9 mRNA in peritoneal and alveolar macrophages and macrophage‐rich organs (lung and bone marrow), whereas in non‐Tg rabbits, endogenous MMP‐9 mRNA expression was extremely low and only detected in the bone marrow, presumably in bone marrow macrophages (Figure 1C). To analyse MMP‐9 proteins and enzymatic activity, we isolated elicited macrophages from the peritoneal cavity of rabbits. On Western blotting analysis of the conditioned media, the peritoneal macrophages from Tg rabbits secreted approximately 10‐fold more (without PMA treatment) and fourfold more (after PMA treatment) MMP‐9 protein than control macrophages, as calculated by OD values (Figure 1D). Gelatin zymography demonstrated that MMP‐9 proteins secreted by macrophages are enzymatically active (Figure 1D).

### 3.2 Effects of MMP‐9 on the initiation of atherosclerosis

**Figure 2:**
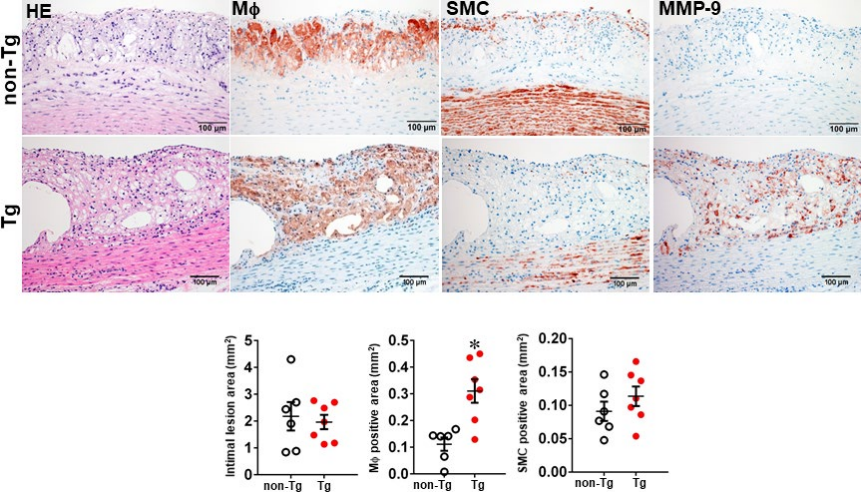
Comparison of histological features of early‐stage atherosclerotic lesions. Male Tg and non‐Tg rabbits were fed a cholesterol diet for 16 weeks, and the aortic lesions were then quantified microscopically. Representative micrographs of the aortic arch lesions are shown on the top panel. Serial paraffin sections of the aortic arch were stained with haematoxylin and eosin (HE), or immunohistochemically stained with monoclonal antibodies (mAbs) against either macrophages (Mϕ), or α‐smooth muscle actin for smooth muscle cells (SMC) or MMP‐9. Intimal lesions on EVG‐stained sections and positively stained areas of Mϕ and SMC were quantified with an image analysis system (bottom panel). Values are mean ± SEM, n = 6‐7. *P < .05 vs non‐Tg rabbits

We first examined whether MMP‐9 overexpression affected the early‐stage lesion formation of atherosclerosis in rabbits fed a cholesterol diet for 16 weeks. As shown in Figure S1, both male and female Tg and non‐Tg rabbits developed similar hypercholesterolaemia throughout the experiment. The aortic lesions were mainly found in the aortic arch, and the gross lesion area was not significantly different between Tg and non‐Tg rabbits of both male and female (Figure S2). Histological examinations revealed that the lesions of aortic atherosclerosis in Tg and non‐Tg rabbits were predominantly composed of type II lesions (fatty streaks), which were characterized by foam cell accumulation intermingled with small numbers of smooth muscle cells and ECM; however, the number of lesional macrophages was significantly increased by 2.8‐fold in male (Figure 2) and 1.3‐fold in female Tg rabbits (Figure S3). In these areas, MMP‐9 immuno‐reactive proteins were co‐localized with macrophages in Tg rabbits, whereas MMP‐9 was barely stained in the lesions of non‐Tg rabbits (Figure 2 and Figure S3).

### 3.3 Effects of MMP‐9 on the progression of atherosclerosis

**Figure 3:**
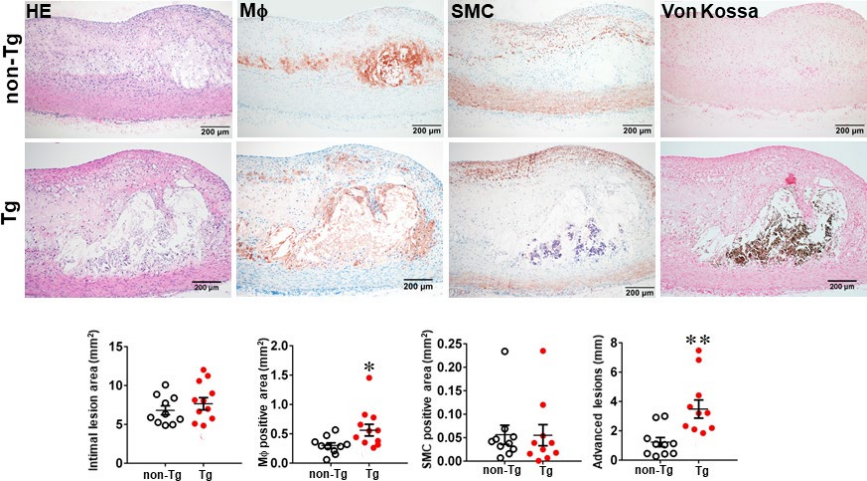
Comparison of histological features of advanced atherosclerotic lesions of aorta. Male Tg and non‐Tg rabbits were fed a cholesterol diet for 28 weeks, and the aortic lesions were then quantified microscopically. Representative micrographs of the aortic arch lesions are shown on the top panel. Serial paraffin sections of the aortic arch were stained with haematoxylin and eosin (HE) and von Kossa or immunohistochemically stained with monoclonal antibodies (mAbs) against either macrophages (Mϕ) or α‐smooth muscle actin for smooth muscle cells (SMC). Intimal lesions on EVG‐stained sections and positively stained areas of Mϕ and SMC in the intima were quantified with an image analysis system (bottom panel). Advanced lesions were measured by calculating the average length of lesions with lipid cores and calcification as described in the Methods. Values are mean ± SEM, n = 10‐12. *P < .05, **P < .01 vs non‐Tg rabbits

In the second experiment, we fed rabbits with a cholesterol diet for 28 weeks to examine whether increased MMP‐9 expression influenced the advanced lesion formation. Compared with the experiment 1 in which the lesions were smaller in size and mainly consisted of the fatty streaks, the lesions of the experiment 2 were more extensive and predominately by advanced lesions, such as fibrous plaques and atheroma (types IV‐V lesions).^40^ The total cholesterol levels of Tg and non‐Tg rabbits were essentially similar (Figure S4) but sudanophilic areas (gross lesions) and total microscopic lesions of the aortic arch were significantly increased in female Tg rabbits compared with non‐Tg littermates, even though no significant difference in male Tg rabbits compared with non‐Tg controls (Figures S5, S6 and Figure 3). Regardless of this gender difference in lesion size, microscopic examinations showed that the advanced lesions (types IV‐V lesions) were increased in both male and female Tg rabbits: threefold greater in male and 2.8‐fold larger in female Tg rabbits than those in non‐Tg rabbits (Figure 3 and Figure S6). In contrast to the fatty streaks which are enriched in macrophages and foam cells, advanced lesions were characterized by necrotic or lipid cores covered by a layer of fibrotic cap and were frequently associated with marked calcium deposition or calcification (Figure 3). In addition, macrophage staining area was significantly increased by 1.9‐fold in male and 1.6‐fold in female Tg rabbits compared with non‐Tg rabbits whereas SMC components in the lesions were unchanged between Tg and non‐Tg rabbits (Figure 3 and Figure S6).

**Figure 4:**
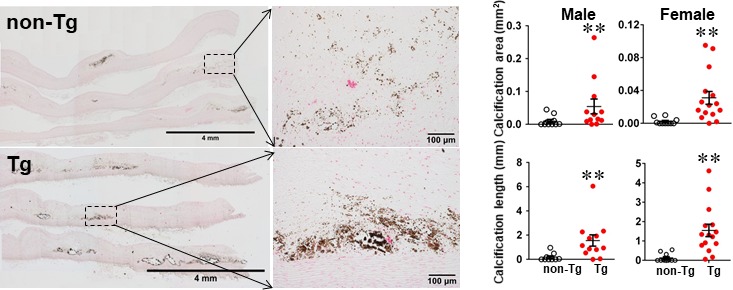
MMP‐9 expression increases aortic calcification in Tg rabbits. The aortic arch sections (8‐10 sections for each animal) were stained with von Kossa stain (A), and both the calcified area and length on the sections were measured. Values are mean ± SEM, n = 10‐16. Each dot represents the data of an individual animal. *P < .05, **P < .01 vs non‐Tg rabbits

**Figure 5:**
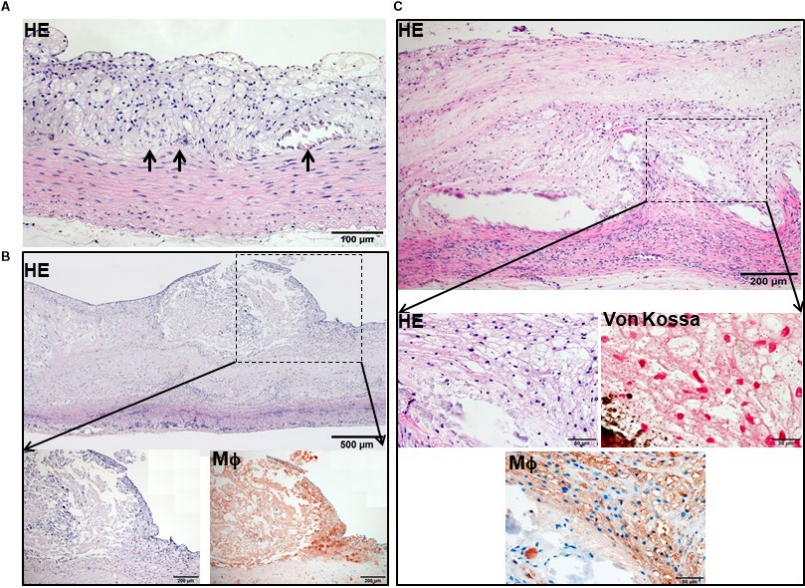
Different microscopic patterns of aortic calcification in Tg rabbits. Calcified materials were observed in the centre of fatty streak type lesions (possibly associated with apoptotic macrophages) (the arrows point the calcified area) (A). Calcification was associated with fragile or unstable lesions which have a large lipid core and a thin cap (B). (Note: the surface of the lesions looks ‘erupted’ possibly caused by artefact during the specimen preparation). Calcium particles (~1 μm) within macrophage accumulation are possibly those of so‐called microcalcification‐generating matrix vesicles (von Kossa staining on the right) (C)

**Figure 6:**
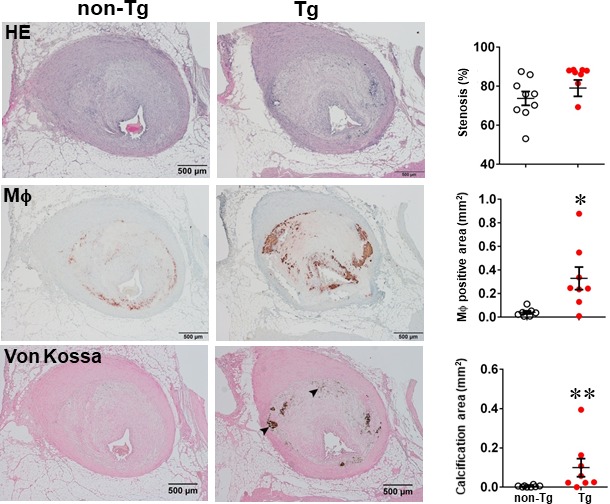
MMP‐9 expression increases coronary calcification in Tg rabbits after feeding a cholesterol diet for 28 weeks. Serial paraffin sections of left coronary artery lesions of male non‐Tg and Tg rabbits were stained with H&E, mAb against macrophages or von Kossa. The coronary stenosis = lesion area/total lumen area × 100(%) was measured and is expressed as a percentage, and the macrophages (Mϕ) positive area and calcification area in lesions were quantified. Data are expressed as mean ± SEM, n = 8 for each group. Each dot represents the data of an individual animal. *P < .05, **P < .01 vs non‐Tg rabbits

One of the striking features of advanced lesions observed in Tg rabbits was the prominent vascular calcification (Figure 4). Although calcifications are not specific in the lesions of atherosclerosis in cholesterol‐fed Tg rabbits, 98% of Tg rabbits (26/27) showed severe calcified lesions associated with atherosclerosis, whereas only 40% (8/20) of non‐Tg rabbits had calcified lesions as observed under light microscopy using HE and von Kossa stained specimens. In addition to the higher prevalence of vascular calcification in Tg rabbits, average calcified areas along with calcified length on the sections stained by von Kossa staining in the aortic lesions of Tg rabbits were much larger than those in non‐Tg rabbits: 5.6‐fold greater in males and 12.9‐fold greater in females (Figure 4). Calcification was not only found in the deep areas of the lesions such as lipid cores (Figure 4) but also observed in the fatty streaks where many macrophage‐derived foam cells (presumably those of apoptotic macrophages as shown below) were present (Figure 5A). In some areas, calcified lesions of Tg rabbits showed apparent ‘unstable’ vulnerable features such as large lipid core with a thin fibrotic cap accompanied by macrophage accumulation in the shoulders (Figure 5B). In macrophage‐rich regions, we could also observe many so‐called microcalcification‐generating matrix vesicles (Figure 5C). Many lesional macrophages associated with calcification were assumptively apoptotic because they could be stained by both TUNEL and caspase‐3 staining (Figure S7). Interestingly, marked calcification was also found in coronary arteries: 21.6‐fold increase of calcification areas in male Tg rabbits (Figure 6) and 12.9‐fold increase in female Tg rabbits (Figure S8). In these calcified areas, marked macrophage accumulation was often observed: 8.1‐fold increase in male Tg rabbits and 2.5‐fold increase in female Tg rabbits compared with non‐Tg rabbits. Coronary stenosis was increased in male Tg rabbits even though not significantly different (ns).

In addition to the severe advanced lesions associated with prominent calcification in Tg rabbits described above, macrophages frequently expanded the deep parts of the tunica media or even the adventitial layers (Figure S9).

### 3.4 Expression of MMPs and their endogenous inhibitors in lesions

**Figure 7:**
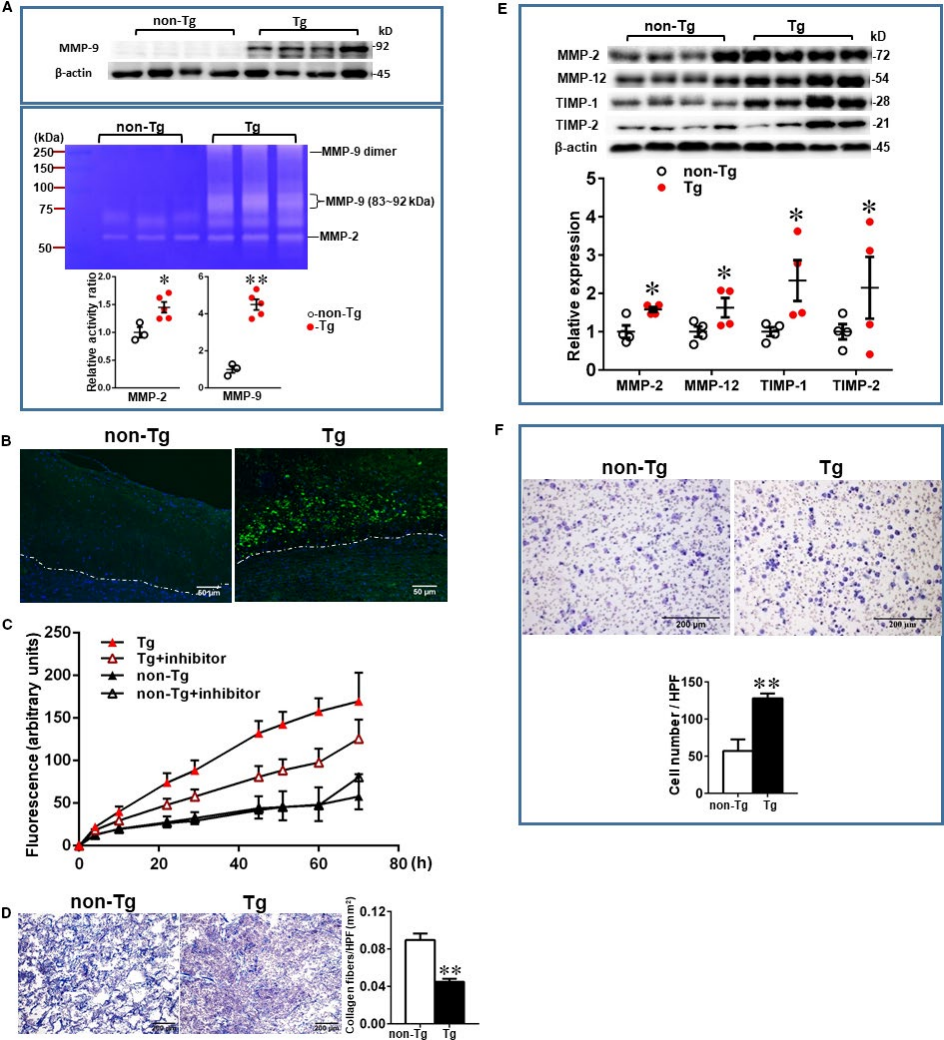
Demonstration of increased MMP‐9 expression in the lesions of Tg rabbits. Proteins isolated from aortic samples were fractionated on 10% SDS‐PAGE and immunoblotted with MMP‐9 and β‐actin was used as an internal control (A, top). Zymography was performed using gelatin as substrate as described in the Methods (A, bottom). n = 3 for each group. To measure the gelatinase enzymatic activity directly in the lesions, we performed in situ zymography as described in the Methods. The gelatinase activity on the sections of aortic lesions was visualized as green, and nuclei were stained as blue (B). White dotted lines indicate the internal elastic lamina. For quantification of gelatinase activity, the homogenate of aortic arch was mixed with quenched fluorescein‐conjugated gelatin, and fluorescent density was measured and expressed as the gelatinase activity (C). High gelatinase activity of Tg rabbits was inhibited in the presence of MMP‐2/9 inhibitors (C). Values are mean ± SEM, n = 4 for each group. Carrageenan‐induced granuloma sections were prepared as described in the Methods (D). Paraffin sections of granulomas were stained by Masson trichrome staining, and the positive areas for collagen fibres (blue or purple) were quantified with an image system (D). Ten high‐power fields (HPF) of each section were randomly selected and calculated. Data are expressed as mean ± SEM, n = 4 for each group. **P < .01 vs non‐Tg rabbits. Proteins isolated from aortic arch were fractionated on 10% SDS‐PAGE and immunoblotted with each mAb against MMP‐2, MMP‐12, TIMP‐1 TIMP‐2 and β‐actin (E). Data are expressed as mean ± SEM and n = 4 for each group. Alveolar macrophage chemotaxis assays were performed as described in the Methods. Results are expressed as the average number of cells that migrated through immobilized ECM film after 48 h incubation (F). Data are expressed as mean ± SEM and n = 4 for each group. **P < .01 vs non‐Tg rabbits

To examine MMP‐9 expression along with other MMPs and TIMPs in the lesions, we further quantified the content of MMP‐2, MMP‐9, MMP‐12 and TIMP‐1 and TIMP‐2. In compatible with the finding shown by immunohistochemical staining (Figure 2 and Figure S3), increased expression of MMP‐9 proteins were further confirmed by Western blotting and zymography (Figure 7A). Low expression of MMP‐9 in the lesions of hypercholesterolemic rabbits was also reported in the previous study.^45^ High levels of MMP‐9 expression of the lesions in Tg rabbits were associated with incremental gelatin‐degrading activity as shown by gel zymographic analysis, in situ zymography, and gelatinase activity assay (Figure 7B,C). Gelatinase activity in aortic lesions visualized by in situ zymography can be inhibited in the presence of EDTA (data not shown). Furthermore, we found that carrageenan‐induced granulomas of Tg rabbits contained significantly less extracellular matrix content (47% decrease, *P* < .01) than those of non‐Tg rabbits (Figure 7D). In addition, Western blotting showed that increased MMP‐9 was actually accompanied by significant increase of MMP‐2, MMP‐12, TIMP‐1 and TIMP‐2 in Tg rabbits (Figure 7E).

### 3.5 MMP‐9 enhances macrophage migration and digestion of extracellular matrix

To clarify the mechanisms responsible for increased macrophage accumulation in the lesions of Tg rabbits, we compared the capacity of alveolar macrophages isolated from Tg and non‐Tg rabbits to invade an immobilized ECM in vitro. In response to the presence of the chemoattractant MCP‐1, the number of gel‐invading macrophages from Tg rabbits was 2.3‐fold greater than that from non‐Tg rabbits (*P* < .01) (Figure 7F).

## 4 DISCUSSION

Accumulation of macrophage‐derived foam cells in the intima of large arteries is a hallmark of atherosclerosis. Macrophages secrete a variety of bioactive substances, such as MMPs and cytokines, thereby playing an important role in lesion formation and progression.^32^ In the current study, we focused on MMP‐9, an important gelatinase, and generated Tg rabbits that overexpressed MMP‐9 in the macrophage lineage to examine whether increased MMP‐9 expression affects the initiation and progression of atherosclerosis. For this undertaking, we fed Tg and non‐Tg rabbits a cholesterol diet for 16 and 28 weeks.

In the first experiment, we did not see any significant influence of MMP‐9 expression on the fatty streak size but the macrophage number was indeed increased in the lesions of Tg rabbits. In this aspect, MMP‐9 is similar to other MMPs such as MMP‐1 (collagenase) and MMP‐12 (elastase) because Tg rabbits expressing either MMP‐1^23^ or MMP‐12^47^ in macrophages also failed to increase the fatty steak formation. Meanwhile, macrophage lineage overexpressing MMP‐9 in apoE KO mice showed no effects on lesion size.^41^ It has been reported that MMP‐9 deficiency appears to enhance plaque growth in apoE/MMP‐9 double KO mice^30^ although MMP‐9 deficiency protected against cholesterol diet‐induced atherosclerosis in the same double KO mice.^29^

In the second experiment, rabbits were fed a cholesterol diet for 28 weeks and developed greater and more advanced lesions (than experiment one) which enabled us to examine whether increased MMP‐9 affects the progression of atherosclerosis. Although the aortic arch lesions stained by Sudan IV became almost saturated at 28 weeks, gross lesion areas were significantly increased in female (not male) Tg rabbits. The molecular mechanism for this gender effect of MMP‐9 is still unknown but it will be interesting to investigate whether sex hormones interact with MMP‐9 in future. Nevertheless, lesional macrophages were significantly increased in both aortic and coronary atherosclerotic lesions in both male and female Tg rabbits compared with non‐Tg rabbits*.* Accumulation of macrophages in the lesions even extended to the deeper medial tunica or even the adventitia. Although it is still unknown whether these adventitial macrophages come from the inside or outside, it is most likely that they develop as a result of ‘outside in’ rather than ‘inside out’ migration. Regardless of this assumption, emigration of monocytes/macrophages requires the degradation of the ECM and increased MMP‐9 activity in Tg macrophages undoubtedly leads to enhancement of ECM degradation thereby facilitating the influx of monocyte/macrophages into the lesions.^28^ This contention was further supported by our chemotaxis study and granuloma model assay showing that macrophages from Tg rabbits exhibited higher infiltrative activity towards a chemoattractant, MCP‐1 in vitro and augmented capability of hydrolysing ECM in granulomatous tissue (Figure 7). Previous studies using apoE KO mice demonstrated that local expression of catalytic form of MMP‐9 by adenoviral vectors enhances plaque rupture and haemorrhage.^16^, ^48^ In the current study, we observed so‐called “vulnerable sites” in the aortic lesions of Tg rabbits but did not find any plaque ruptures, suggesting pro‐MMP‐9 activation is a key process of MMP‐9‐derived plaque rupture.

In spite of this, one of the most striking findings observed in the current study was the demonstration of remarkable calcification in the aortic and coronary lesions in Tg rabbits, which mimicked human advanced atherosclerotic lesions with calcification.^17^, ^49^ This finding was initially unexpected and surprising, but it is possible that MMP‐9 may mediate vascular calcification through several possible molecular mechanisms.^50^, ^51^ First, it has been reported that apoptotic macrophages in the lesions can release microcalcification‐generating matrix vesicles into the ECM, eventually resulting in calcification^52^ which is supported by our finding that macrophage‐rich lesions contained microcalcification. High expression of caspase‐3 in the lesions of Tg rabbits allowed us to speculate that increased cell death may be also involved in the process of vascular calcification. Given the fact that overexpression of MMP‐9 led to the incremental infiltration of macrophages in the lesions of Tg rabbits, it is presumable that apoptotic macrophages would be increased. It is not clear; however, whether increased MMP‐9 expression changes the calcium milieus in the intima, or stimulates macrophages to release more microcalcification‐generating matrix vesicles.^53^ Second, it is well known that macrophages can differentiate into osteoclasts that help the active resorption of the ECM in the arterial wall. If this process was retarded, calcium deposition in the lesions would be enhanced. This notion was supported by the observation that TRAP staining, a histochemical marker for osteoclast differentiation^54^ were significantly reduced in atherosclerotic lesions of Tg rabbits compared with non‐Tg rabbits (Figure S10). Finally, besides macrophages, vascular smooth muscle cells constitute another cellular source that participates in the vascular calcification. For example, increased MMP‐9 can lead to incremental hydrolysis of elastin and such soluble elastin‐derived peptides from MMP‐9 hydrolysis promote osteogenic differentiation of SMCs in arterial wall.^55^ In addition to MMP‐9, the lesional MMP‐2 expression was simultaneously increased in Tg rabbits possibly caused by increased macrophages in the lesions, which may also affect lesion development and vascular calcification.^56^, ^57^ It should be pointed out that in addition to MMPs, cathepsins or other proteinases in the lesions may be also involved in the lesion development.^58^, ^59^ It will be interesting to investigate how these proteinases play an interactive role in the development of atherosclerosis. Nevertheless, vascular calcification in the atherosclerotic lesions apparently affects arterial stiffness and contractility thus impairing blood flow which leads to ischaemia in the organs. Our current results may support the notion that inhibition of vascular macrophage‐derived MMP‐9 may become a new potential therapeutics to prevent vascular calcification,^60^, ^61^ while this hypothesis remains to be verified in future.

In conclusion, we have successfully created Tg rabbits that overexpress MMP‐9 specifically in macrophage lineage. Increased expression of MMP‐9 in macrophages not only enhances the formation of advanced atherosclerotic lesions in Tg rabbits, but also leads to a marked vascular calcification. Therefore, macrophage‐derived MMP‐9 in the arterial wall may exert other physiological functions beyond its classical gelatinase activity. Although the molecular mechanisms remain yet to be clarified, it will be interesting to investigate in the future whether inhibition of MMP‐9 functions as a therapeutic method to inhibit vascular calcification.

## CONFLICT OF INTEREST

None.

## AUTHOR CONTRIBUTIONS

JF and Y.EC designed the study; KN, SK and FM created and bred Tg rabbits. YC, ABW, BN, LC, HS, TK, YY, JZ, JL and EL performed experiments, collected, analysed and interpreted the data. YC and JF wrote the paper. All authors had final approval of manuscript submission.

## Data Availability

The data used to support the findings of this study are available from the corresponding author upon reasonable request.
