# Supplementary material for: Macrophage‐derived MMP‐9 enhances the progression of atherosclerotic lesions and vascular calcification in transgenic rabbits
Source: J Cell Mol Med. 2020 Mar 3;24(7):4261–74. doi: 10.1111/jcmm.15087 (PMC7171347; doi:10.1111/jcmm.15087)
Supplement: Supplementary file 1 [file JCMM-24-4261-s001.pdf]

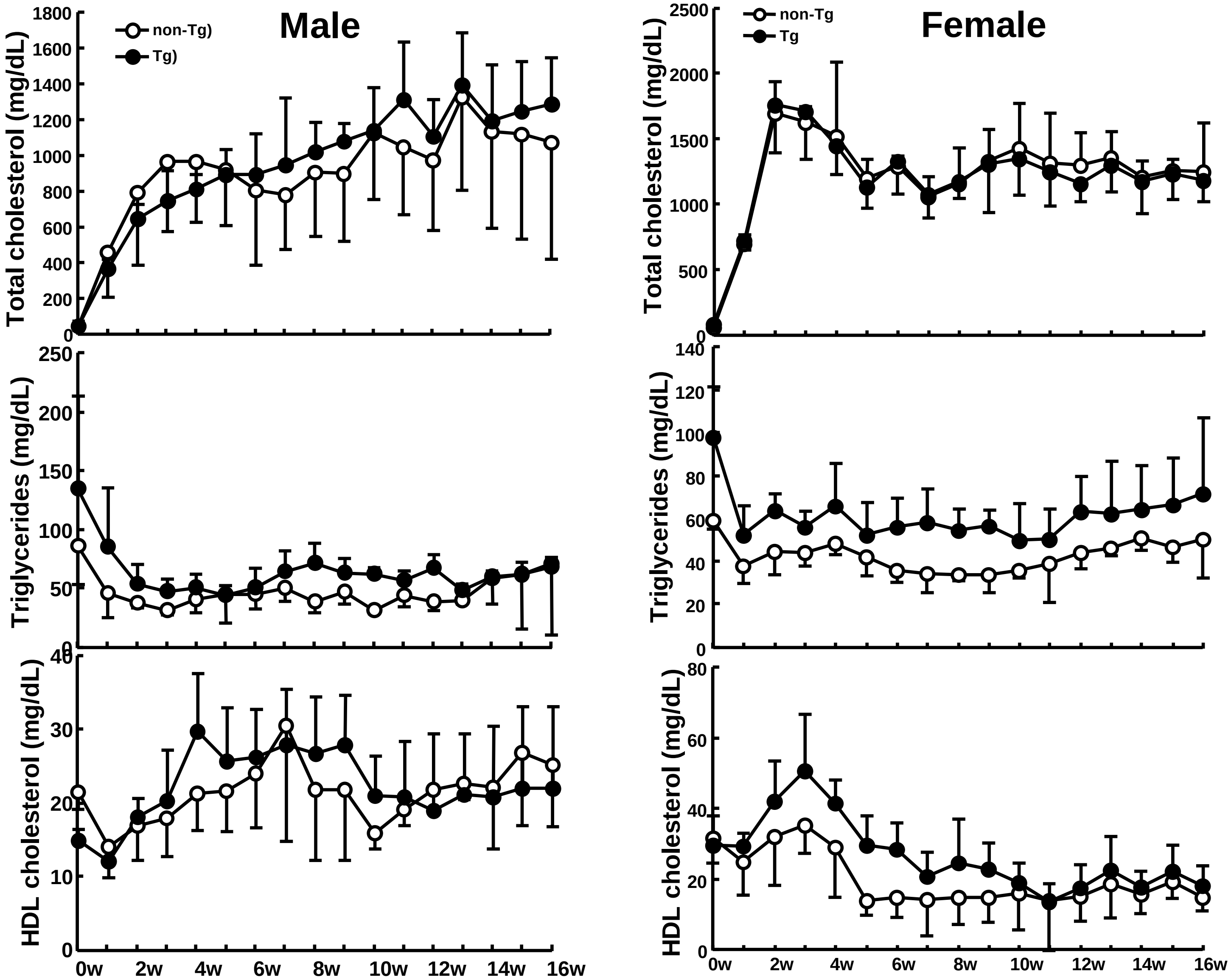

Figure S1. Plasma lipid levels of rabbits fed a cholesterol diet for 16 weeks. Total cholesterol (TC), triglycerides (TG) and high density lipoprotein (HDL)-C levels in plasma of male (left) and female (right) rabbits. There are no significant differences in plasma lipids between Tg and non-Tg rabbits. Data are expressed as mean ± SEM and n=4-8 for Tg and non-Tg rabbits.

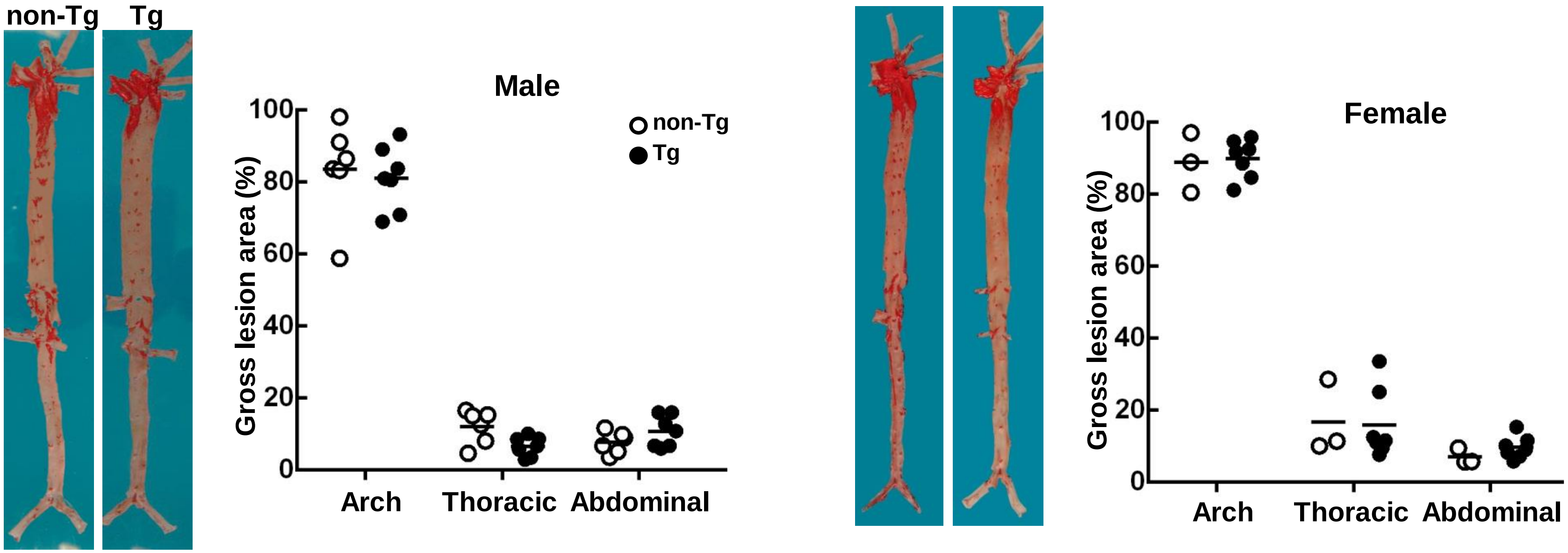

Figure S2. Comparison of gross aortic atherosclerosis lesions of male and female Tg and non-Tg rabbits fed a cholesterol diet for 16 weeks. Representative pictures of aortas stained with Sudan IV from each group are. The lesion area (defined by the sudanophilic area) was quantified using an image analysis system. Each dot represents the lesion area of an individual animal.

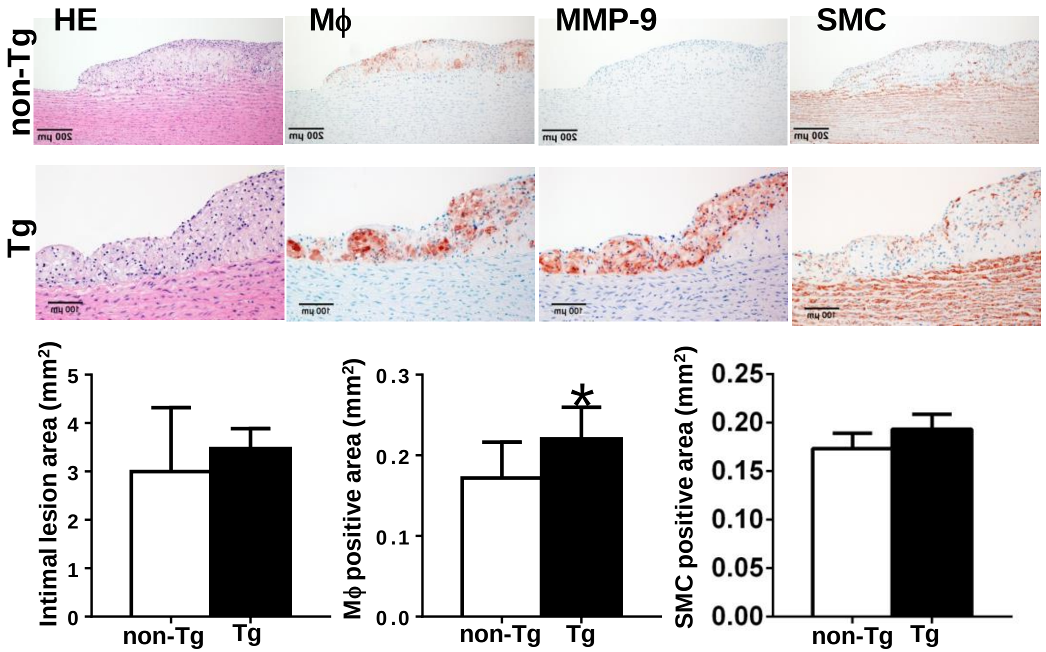

Figure S3. Histological features of early-stage atherosclerotic lesions in female group after a cholesterol diet for 16 weeks were shown. Serial paraffin sections of aortic arch were stained with HE, or immunohistochemically stained with monoclonal antibodies (mAbs) against either macrophages (Mϕ), MMP-9, and α-smooth muscle actin for smooth muscle cells (SMC). Intimal lesions area and positively stained areas of Mϕ and SMC were quantified. Values are mean ± SEM, *n*=3-7.

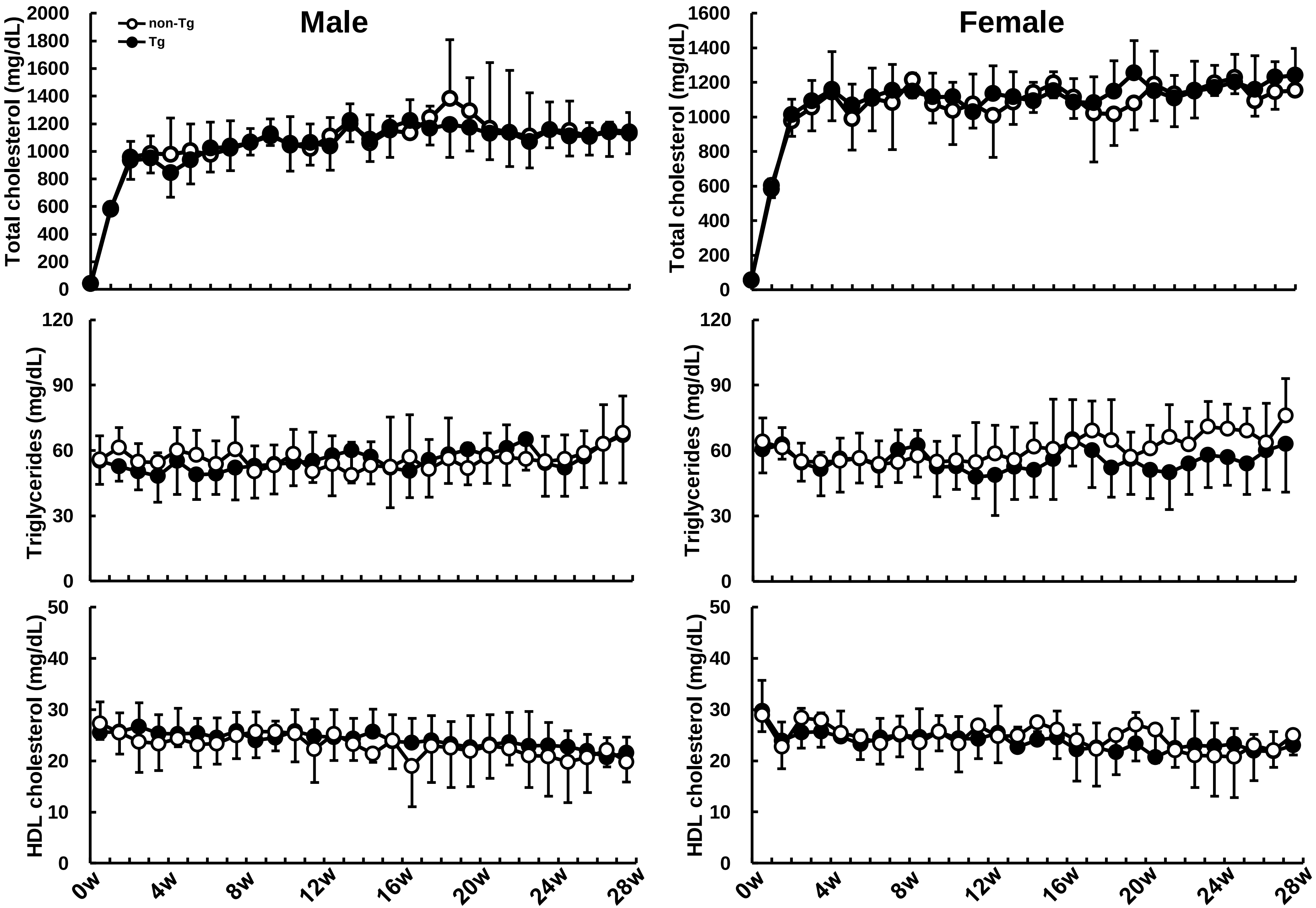

Figure S4. Plasma lipid levels of rabbits fed a cholesterol diet for 28 weeks. Total cholesterol (TC), triglycerides (TG) and high density lipoprotein (HDL)-C levels in plasma of male (left) and female (right) rabbits. Values are mean ± SEM, *n*= 10-12 for male group and *n*=10-16 for female group. There are no significant differences between Tg and non-Tg rabbits.

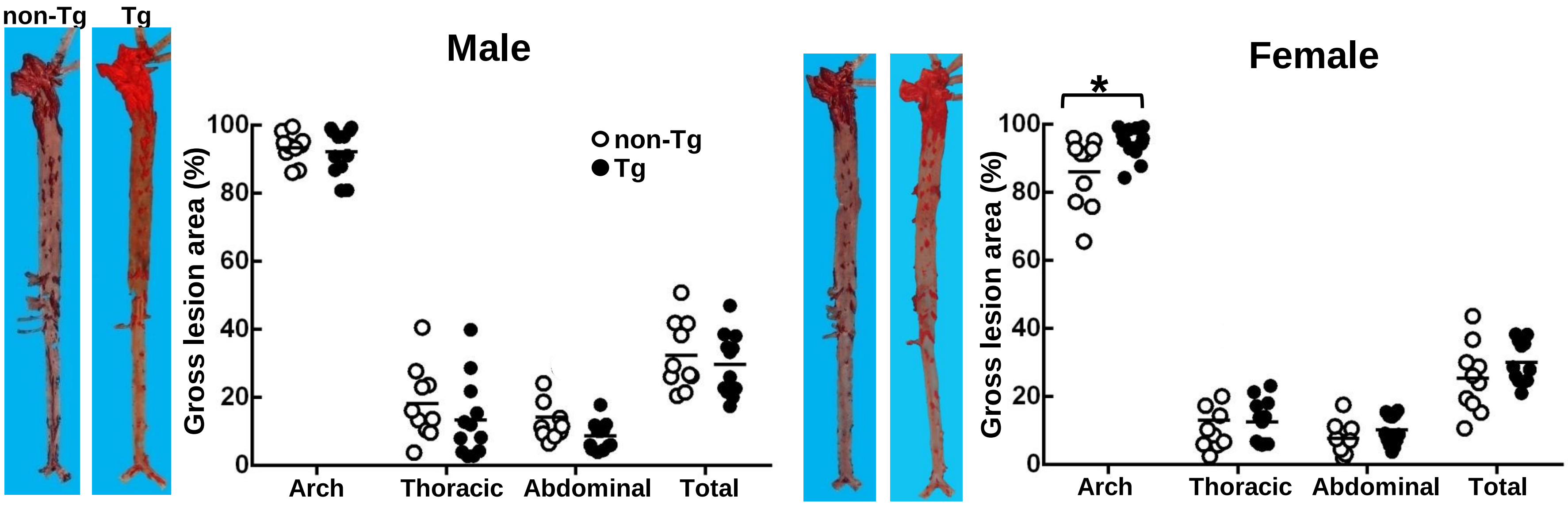

Figure S5. Comparison of gross aortic atherosclerosis lesions of male and female Tg and non-Tg rabbits fed a cholesterol diet for 28 weeks. Representative pictures of aortas stained with Sudan IV from each group are shown. The lesion area (defined by the sudanophilic area) was quantified using an image analysis system. Each dot represents the lesion area of an individual animal. *n*= 10-12 for male group and *n*=10-16 for female group. *p<0.05 vs. non-Tg rabbits.

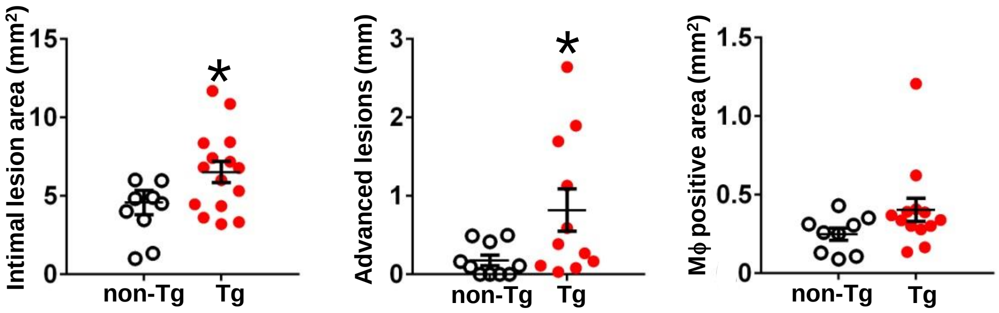

Figure S6. Quantification of microscopic atherosclerotic lesions of aorta of female Tg and non-Tg rabbits. Female Tg and non-Tg rabbits were fed a cholesterol diet for 28 weeks and the aortic lesions were then quantified microscopically. Intimal lesions on EVG-stained sections and positively stained areas of Mϕ were quantified with an image analysis system. Advanced lesions were measured as described in the Methods. Values are mean ± SEM, *n* = 10 for non-Tg and *n*=16 for Tg rabbits. *p<0.05 vs. non-Tg rabbits.

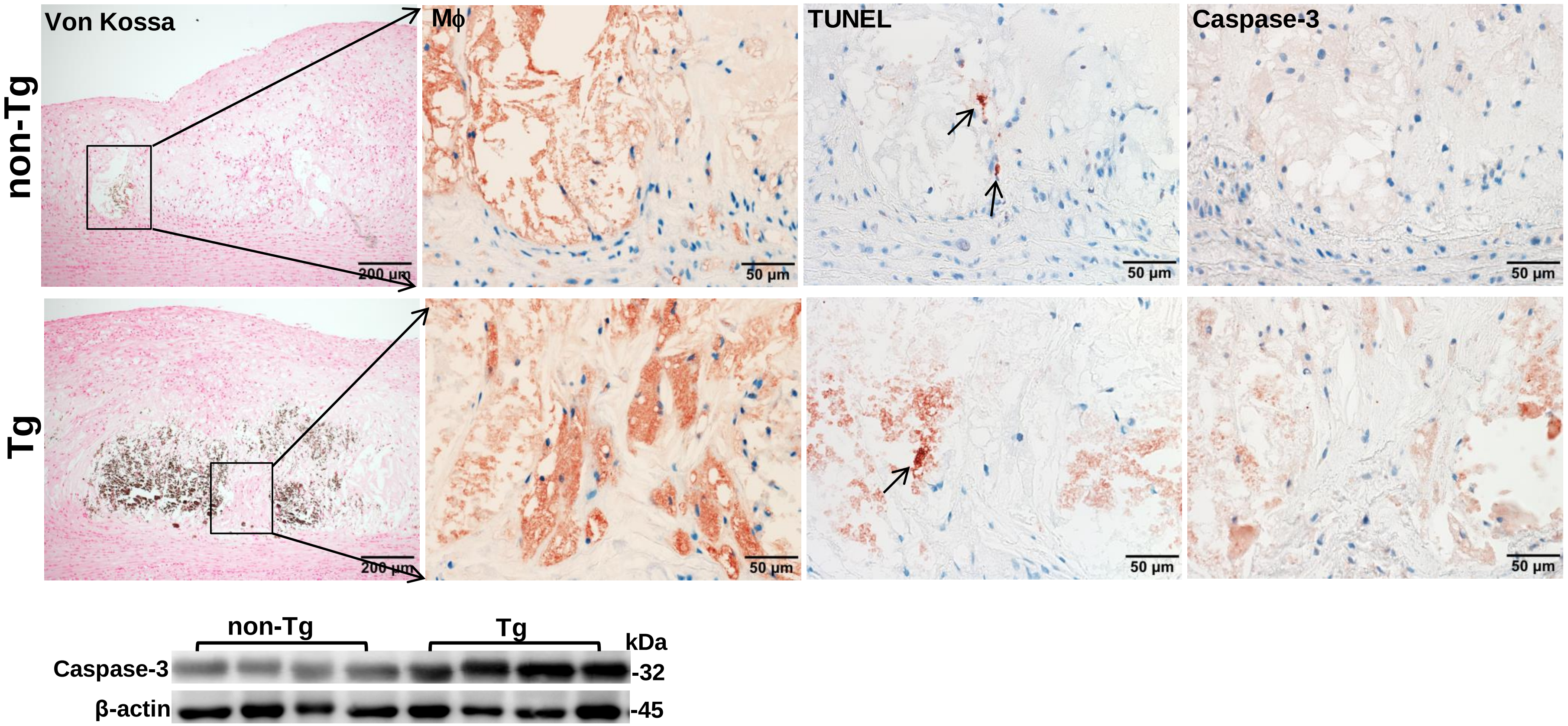

Figure S7. Demonstration of apoptotic cells in the lesions of non-Tg and Tg rabbits.
Serial sections of aortic arch from Tg and non-Tg rabbit were stained Von Kossa and TUNEL or immunohistochemically stained with macrophage (Mϕ) and caspase-3. In necrotic core, TUNEL positive staining area was colocalized with macrophage and caspase-3. Proteins isolated from aortic arch were fractionated on 10% SDS-PAGE and immunoblotted with each mAb against Caspase-3 and β-actin.

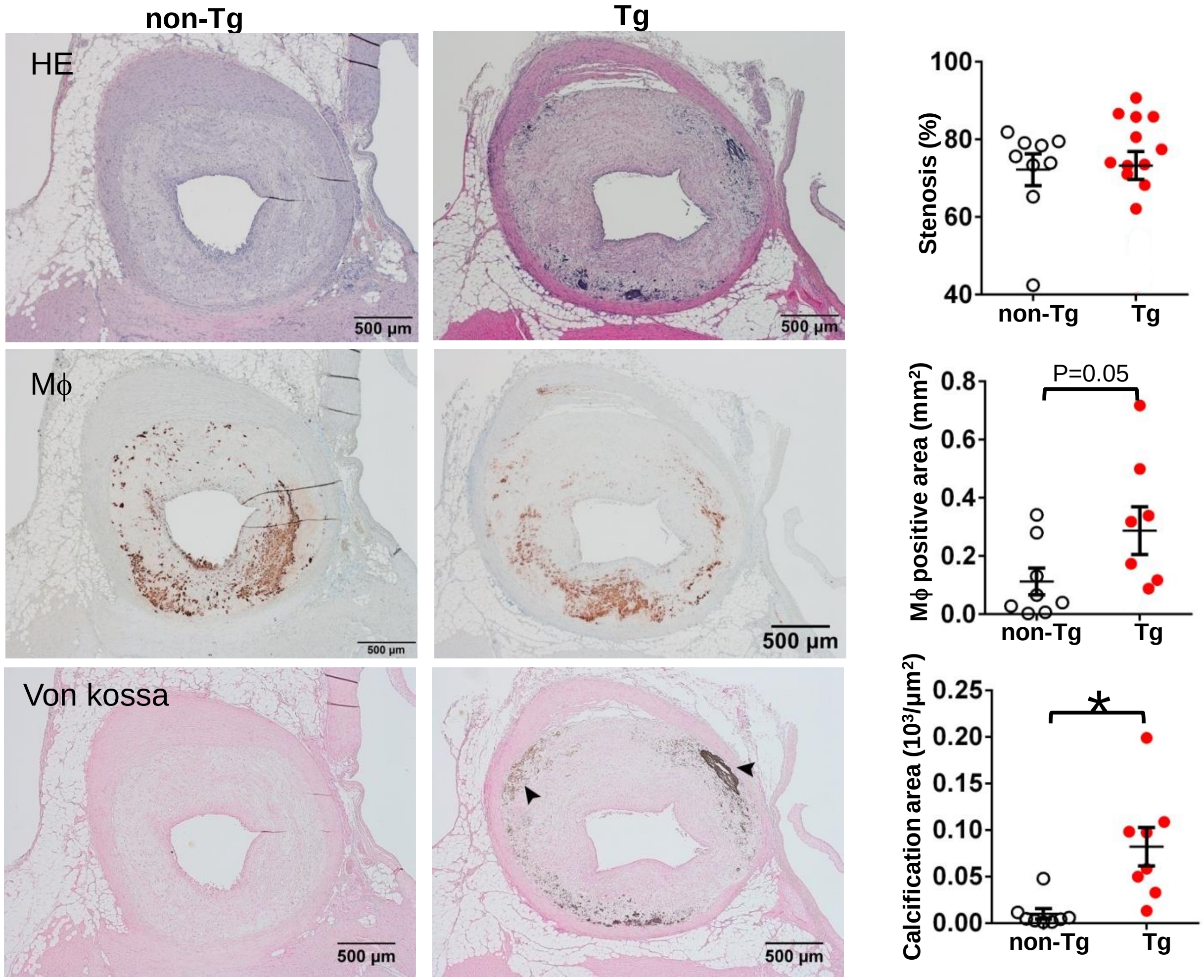

Figure S8. MMP-9 expression increases coronary macrophage infiltration and calcification in female Tg rabbits. Serial paraffin sections of left coronary artery lesions of female non-Tg and Tg rabbits were stained with H&E (left, top), mAb against macrophages (left, middle) and von Kossa (left, bottom). The coronary stenosis=lesion area/total lumen area × 100(%) was measured and is expressed as a percentage (right, top), and macrophages (Mϕ) positive area and calcification area in lesions was quantified (right, middle and bottom). Values are mean ± SEM, $n = 8$. *p<0.05.

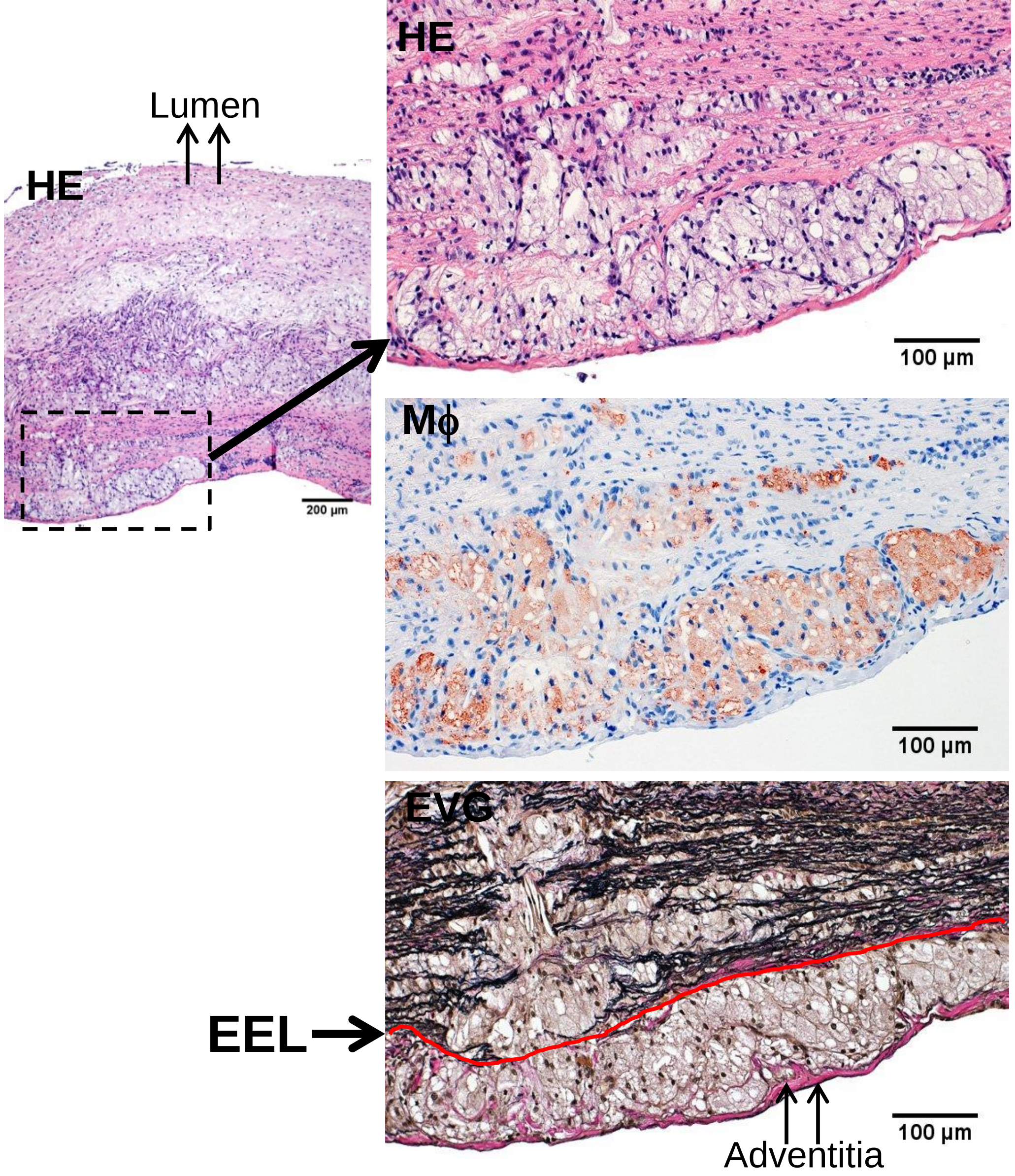

Figure S9 Representative micrographs of aortic adventitial lesions in Tg rabbits. Aortic lesions of Tg rabbits were stained with HE (top), EVG (bottom) or immunohistochemically stained with an mAb against macrophages (middle). Macrophage infiltration expanded the deep parts of the tunica media and the adventitial layers. EEL: external elastic lamina (shown by an arrow).

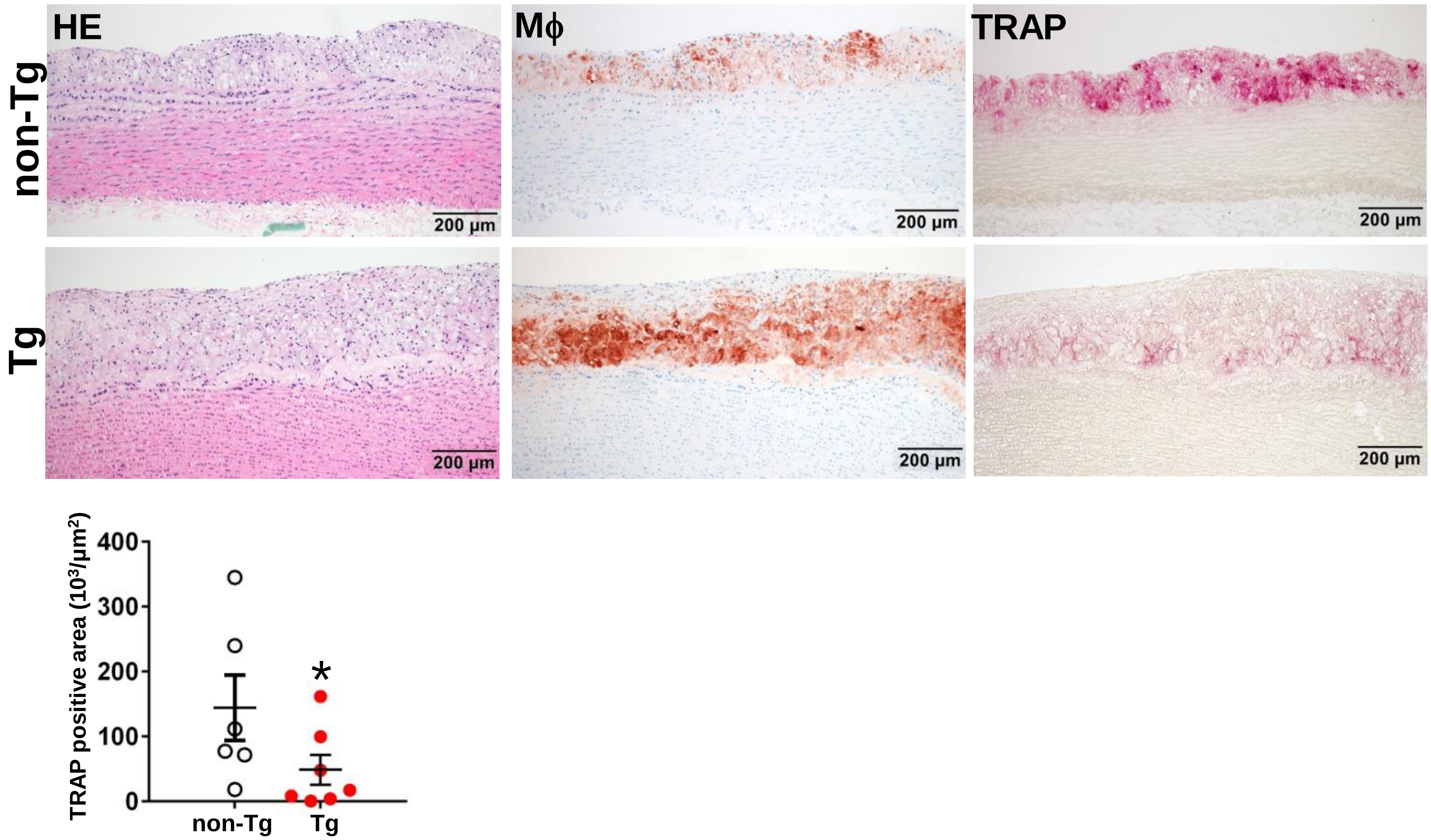

Figure S10. The aortic lesions from Tg and non-Tg rabbits fed a cholesterol diet for 16 weeks were stained with HE, immunohistochemically stained with macrophage (Mϕ) and TRAP staining. TRAP staining intensity was quantified with an image analysis system. Values were mean SEM, n=6-7. *p<0.05 vs. non-Tg rabbits.

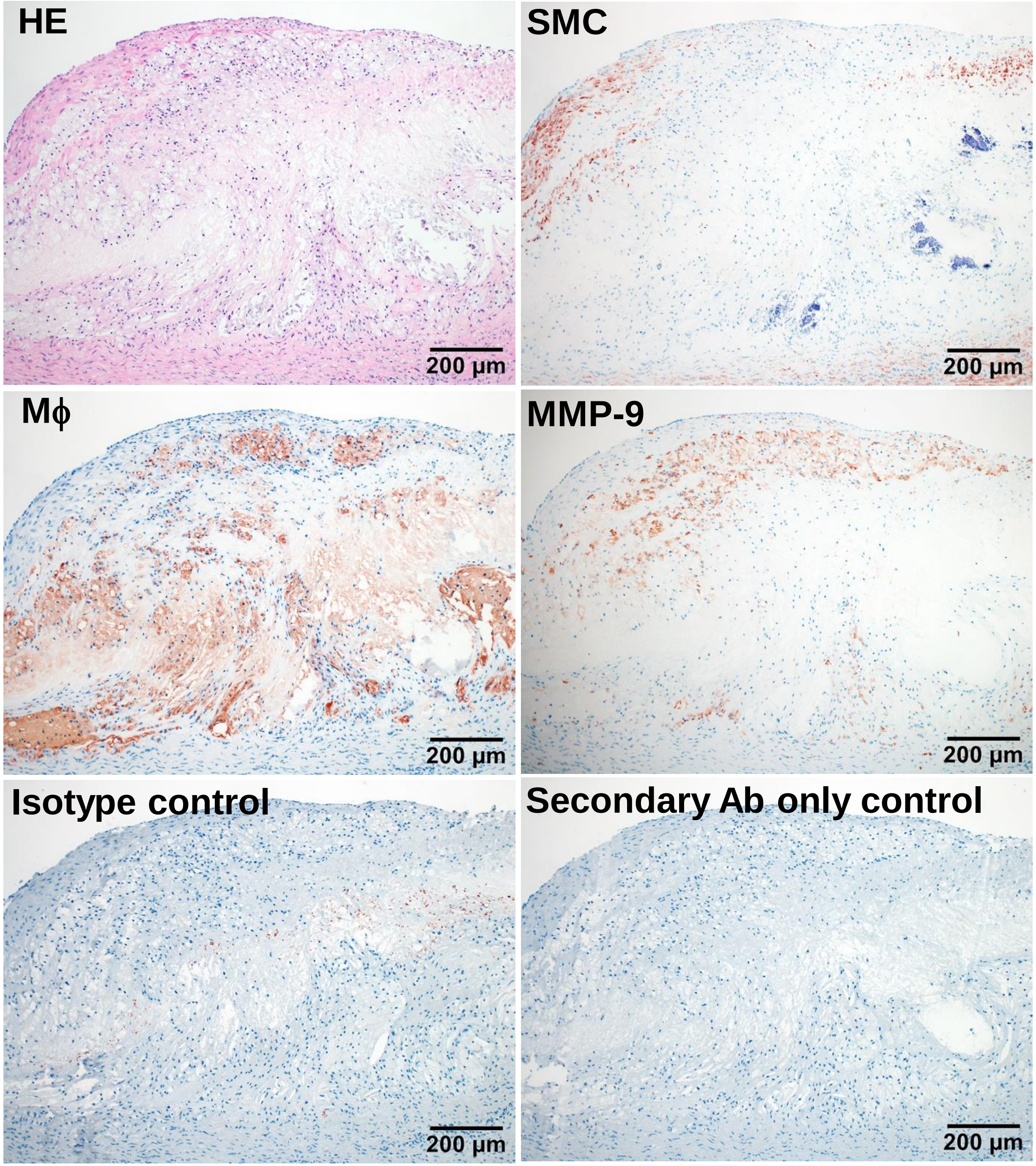

Figure S11. Immunohistochemical staining specificity of antibodies used for the current study. Serial sections of transgenic rabbit aorta were stained with HE and immunohistochemically stained with antibodies against RAM11 (Mϕ), HHF35 (SMC), MMP-9 along with mouse non-specific IgG as the isotype control and secondary antibody only control.

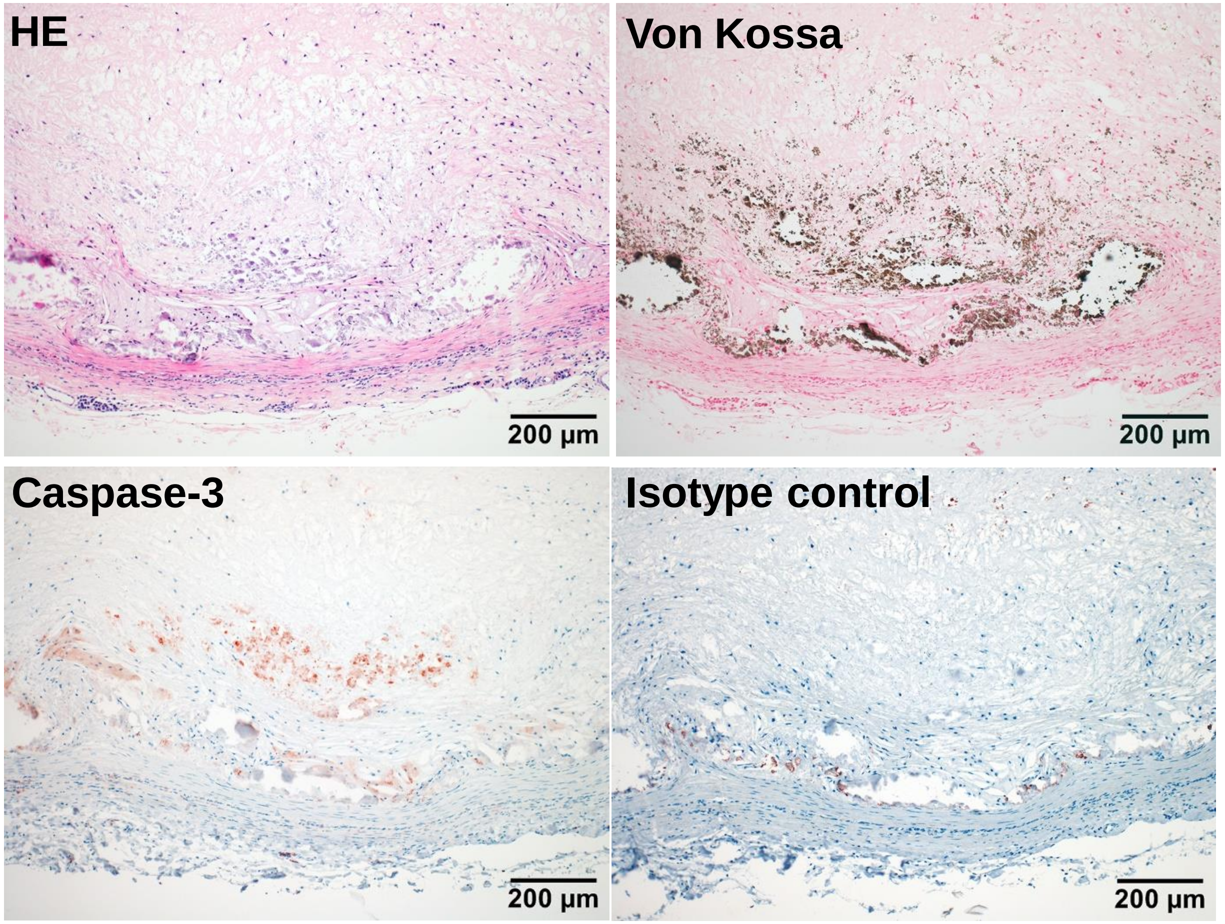

Figure S12. Serial sections of 28 weeks cholesterol-fed rabbit aorta were stained with HE, Von Kossa and immunohistochemically stained with antibodies against caspase-3 along with mouse non-specific IgG as the isotype control. Calcified areas or necrotic areas show somewhat affinity to non-immune mouse IgG.

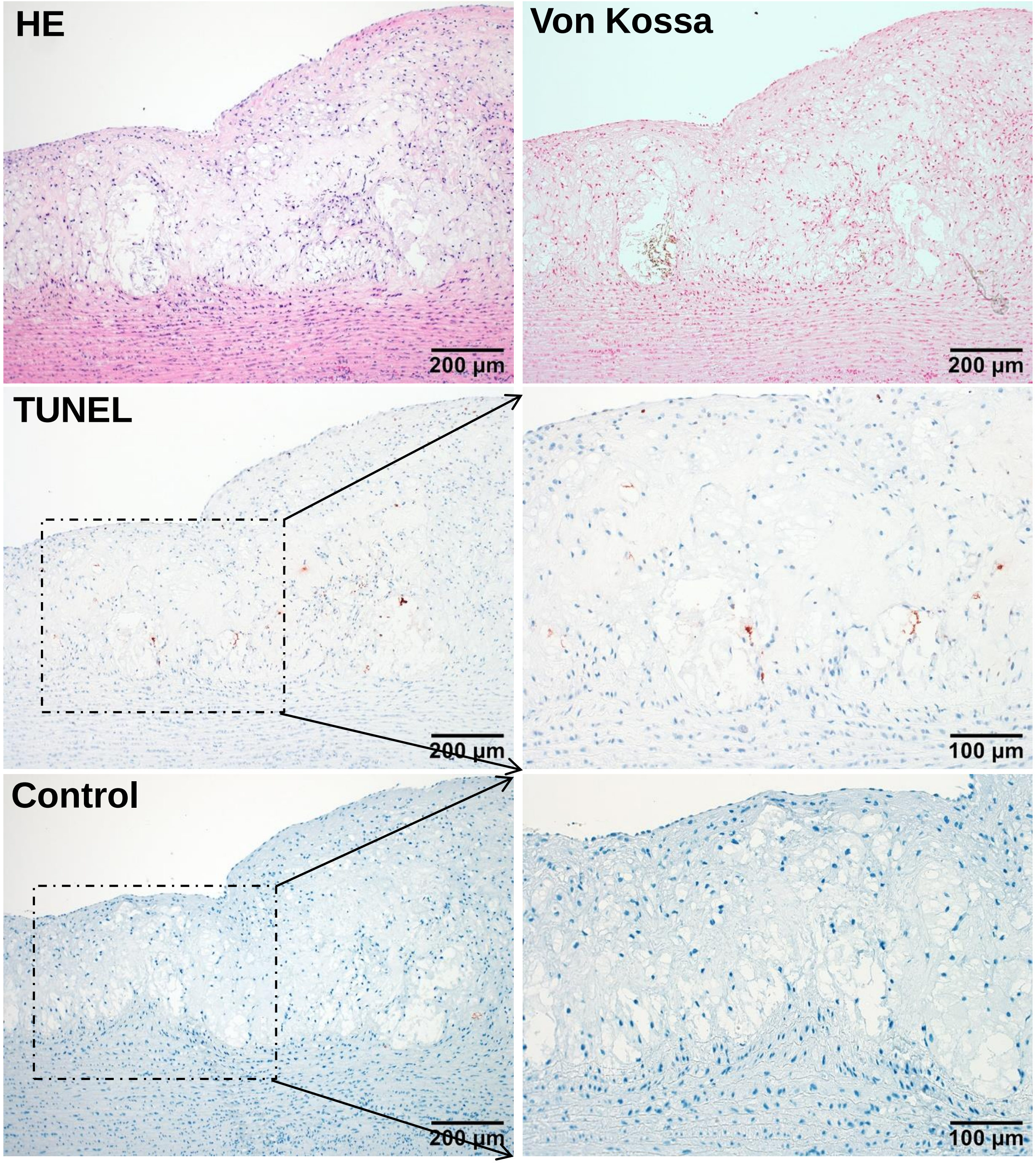

Figure S13. Serial sections of 28 weeks cholesterol-fed rabbit aorta were stained with HE, Von Kossa, TUNEL along with control system. In the control, terminal deoxynucleotidyl transferase (TdT) was replaced by PBS.
