# Supplementary material for: Macrophage‐derived MMP‐9 enhances the progression of atherosclerotic lesions and vascular calcification in transgenic rabbits
Source: J Cell Mol Med. 2020 Mar 3;24(7):4261–74. doi: 10.1111/jcmm.15087 (PMC7171347; doi:10.1111/jcmm.15087)
Supplement: Supplementary file 2 [file JCMM-24-4261-s002.docx]

| Immunoblotting | Dilution | Cat. No. | Manufactures |
| --- | --- | --- | --- |
| MMP-2 | 1:1000 | 42-5D11 | Daiichi Fine Chemical Co., Toyama, Japan |
| MMP-9 | 1:1000 | CL0538 | Sigma-Aldrich Co., St. Louis, MO. |
| MMP-12 | 1:1000 | sc-12361 | Santa Cruz Biotechnology, Inc., Santa Cruz, CA. |
| TIMP-1 | 1:1000 | sc-6832 | Santa Cruz Biotechnology, Inc., Santa Cruz, CA |
| TIMP-2 | 1:1000 | 64-4H11 | Daiichi Fine Chemical Co., Toyama, Japan. |
| β-actin | 1:2000 | sc-81178 | Santa Cruz Biotechnology, Inc., Santa Cruz, CA. |

**Supplemental Table 1A. Antibodies used for western blotting**

| Immunohistochemistry | Dilution | Cat. No. | Manufactures |
| --- | --- | --- | --- |
| Macrophage (RAM) | 1:400 | M0633 | Dako Co., Carpinteria, CA. |
| Muscle actin (HHF35) | 1:300 | M0635 | Dako Co., Carpinteria, CA. |
| MMP-9 | 1:100 | CL0538 | Sigma-Aldrich Co. St. Louis, MO. |
| Caspase-3 | 1:100 | NB100-56708 | Novus Biologicals, Littleton, CO. |
| von Willebrand factor | 1:100 | Abx175155 | Abbexa Co., Cambridge, UK. |

**Supplemental Table 1B. Antibodies used for immunostaining**
